# DFTB Study of Corrosion Inhibitory Properties of (*R*)‑(−) and (*S*)‑(+)-Carvone Isomers

**DOI:** 10.1021/acsomega.5c09679

**Published:** 2026-03-05

**Authors:** Bruno Dantas da Fonseca Souza, Rodrigo Gester, Tarciso Andrade-Filho

**Affiliations:** † Programa de Pós-Graduação em Química, 422009 Universidade Federal do Sul e Sudeste do Pará, Avenida dos Ipês, s/n, Cidade Universitária, Loteamento Cidade Jardim, 68.500-000, Marabá 68508-970, Pará, Brazil; ‡ Faculdade de Física, Universidade Federal do Sul e Sudeste do Pará, Avenida dos Ipês, s/n, Cidade Universitária, Loteamento Cidade Jardim, 68.500-000, Marabá 68.500-000, Pará, Brazil; § Companhia Sinobrás, Rodovia PA-150, Km 425, s/n°, Distrito Industrial, 68508-970 Marabá, Pará, Brazil

## Abstract

Using density functional tight binding (DFTB) calculations, we investigate, for the first time, how molecular chirality modulates the corrosion-inhibition behavior of carvone isomers, the *R*- and *S*-isomers, to evaluate their potential as green organic inhibitors. We assess the isomers with respect to their interactions with the α-Fe(110) surface under various environmental conditions. Adsorption energies and geometries, density of states, and charge distribution are analyzed to describe the adsorption-driven inhibition mechanism. The adsorption of the isomers on the α-Fe(110) surface is driven by van der Waals, electrostatic, and chemical interactions. The results reveal that adsorption is governed by a combination of chemisorption and physisorption, mediated by the carbonyl oxygen and π-electron system of the cyclohex-2-enone ring. The (*S*)-(+) isomer exhibits a greater corrosion inhibition capacity due to more favorable adsorption energies. The explicit inclusion of solvent water molecules during the simulations does not destabilize the inhibitor–surface interaction. These results indicate that the protective layer remains stable under hydrated conditions. These results suggest that (*R*)-(−) and (*S*)-(+)-carvone are promising candidates for sustainable corrosion inhibitors, with (*S*)-(+)-carvone exhibiting superior adsorption stability.

## Graphical abstract

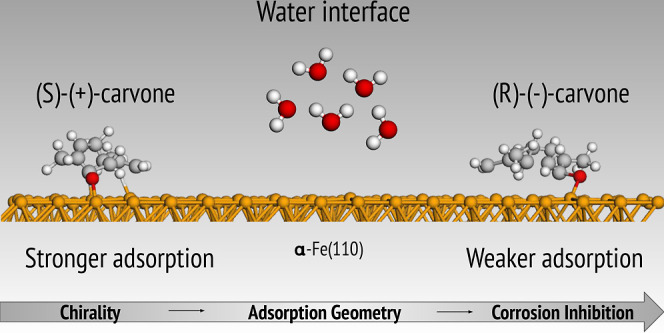

## 1 Introduction

According to the World Corrosion Organization, global expenditure on corrosion is estimated at approximately 2.2 trillion US dollars trillion, representing approximately 3% of GDP.^1^ Iron-based materials are widely used in industry due to their favorable mechanical properties and low cost across various industrial branches.^2^

Corrosion is the natural chemical/electrochemical reaction that causes deterioration, primarily in metallic materials, due to environmental factors,^3^ converting them into undesirable, more stable oxide structures.^3^ The issue arises from a loss of mechanical properties in metallic materials, which poses accident risks in industrial processes, including fluid and gas leaks from pipes and hazards resulting from the material’s reduced mechanical strength.^4,5^

Paints, material coatings, protective metals, and corrosion inhibitors are all effective methods for inhibiting corrosion in metallic systems.^6^ Over the years, the use of inorganic inhibitors has been a long-standing approach. These inhibitors pose high application costs and are environmentally harmful owing to their toxicity.^7^ Therefore, it is necessary to develop new corrosion inhibitors to reduce costs and minimize environmental impact. It is essential to note the use of novel green corrosion inhibitors derived from natural sources. They are also cost-effective to produce and more widely available than their nongreen counterparts.^8,9^ The presence of π-electron systems and heteroatoms such as oxygen, phosphorus, and nitrogen in green organic inhibitors has been shown to prevent metal corrosion.^10,11^ The presence of organic systems, specifically monoterpenes, has been identified as a crucial component of the protective effect observed in these inhibitors.^12–14^ Monoterpenes act as corrosion inhibitors by chelating between the aromatic rings and the metallic surface.^9,15,16^ Considering the class of monoterpenes, carvone is a notable compound illustrated in Figure 1. The system under consideration consists of (*S*)-(+)-carvone (scar, Figure 1, left) and (*R*)-(−)-carvone (rcar, Figure 1, right) isomers. The substance in question is derived from a variety of Amazon essential oils^17–19^ and has also been identified in the Amazon as erva-cidreira [*Lippia alba* (Mill.) N. E. Br., Verbenaceae][Bibr ref18] and a fruit known as bacuri.^20^ Carvone is considered a versatile organic chemical compound with several industrial applications. One technological application is its use as a corrosion inhibitor.^21^ Bensabah et al. found that increasing the concentration of carvone on the surface of the metallic systems can provide greater protection against corrosion.^21^ The high anticorrosive activity of carvone on other metallic systems has already been investigated.^22,23^

**Figure 1:**
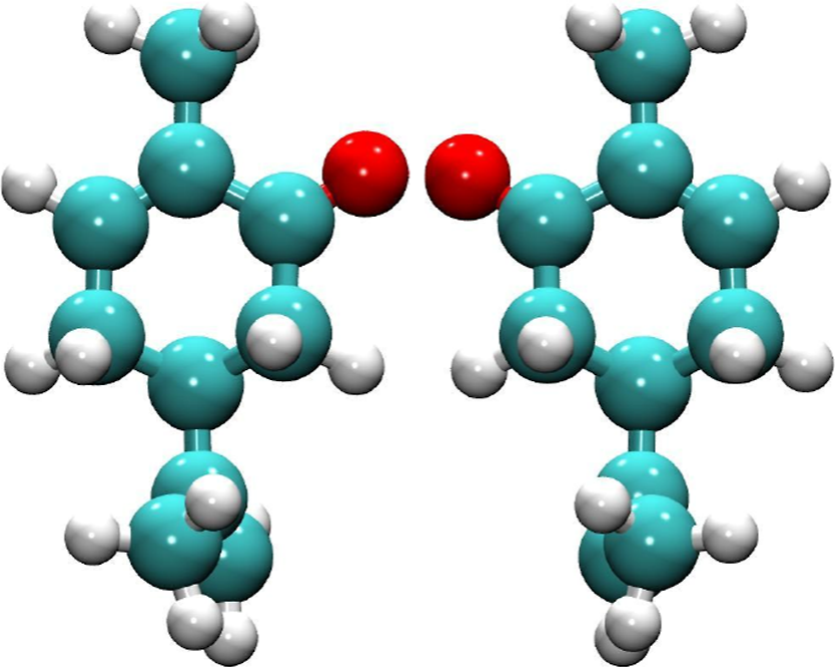
Geometrically optimized structures of scar (left) and rcar (right) isomers.

Identifying the orientation of inhibitor molecules using available experimental techniques alone can be challenging. Consequently, researchers turn to atomistic computer simulations to understand the performance and mechanisms of environmentally friendly, sustainable corrosion inhibitors.^24^ The high computational cost of using the commonly employed Density Functional Theory (DFT) method makes it unfeasible to study large inhibitor-metal systems.^24,25^ Thus, the interaction between the inhibitor-metal system is simulated by adsorbing the investigated isomer molecules onto the iron surface, with calculations performed using the density functional-based tight-binding method with self-consistent charge correction (SCC-DFTB).^26^ The use of this method is motivated by its ability to handle larger metallic surfaces, and the system description is better aligned with experimental observations, thereby adding precision and reducing computational costs compared to DFT calculations.^25^ Moreover, although this method is an approximation, it yields highly accurate results comparable to those obtained by DFT.^26^

This method has been used in corrosion inhibitor applications over the years, namely, Lgaz and Lee used DFTB to study the corrosion inhibition behavior on N80 steel of systems based on the hydrazones (*E*)-2-(4-isobutylphenyl)-*N*′-(4-methoxybenzylidene)­propanehydrazide and (*E*)-*N*′-(furan-2-ylmethylene)-2-(4-isobutylphenyl)­propanehydrazide.^27^ The bond lengths and density of states provided the direction for strong hybridization between the inhibitor atoms and the atoms of the investigated metal surface. Using DFTB, Santos et al. investigated the potential of the two monoterpenes thymol and carvacrol as corrosion inhibitors on the surface (110) of mild steel.^14^ Guo and co-workers investigated the corrosion-inhibition behavior of three chalcones.^24^ The charge-transfer process and the involvement of the systems’ orbitals are fundamental for describing the protection of the investigated surface and, thus, the corrosion-inhibition process at the atomic level.^24^

Therefore, in this work, we use SCC-DFTB to investigate the adsorption of the two carvone isomers on the α-Fe(110) surface, focusing on how these molecules inhibit corrosion and on their interactions with the surface.^24^ Although theoretical atomistic investigations have explored the adsorption of achiral molecules, such as the terpene inhibitors thymol and carvacrol,^14^ on the investigated iron surface, to the best of our knowledge, to date, the influence of molecular chirality on the adsorption mechanism and electronic coupling of terpene-based corrosion inhibitor systems on iron surfaces remains largely unexplored. We present the first theoretical atomistic-based study of the (*R*)-(−)- and (*S*)-(+)-carvone enantiomers on the α-Fe(110) surface in the gas phase while taking into account the influence of an aqueous environment in the calculations to describe the stereochemical effects at the atomic scale.

## 2 Computational Details

In this work, we use the DFTB + package^28^ to perform SCC-DFTB calculations.^26^ Initially, we relax the molecular structures of the investigated monoterpene systems. The crystalline structure of the α-Fe(110) surface iron phase is also relaxed. To achieve this, we relax the iron lattice parameters using data obtained from the experimental crystalline structure.^29^ After relaxing the unit cell, we assemble the α-Fe(110) surface. The assembly process involves four layers in the *c*-axis and a 6 × 6 supercell in the *ab* plane. The Brillouin-zone integrations are performed using a Monkhorst–Pack *k*-point grid of 2 × 2 × 1. The SCC-DFTB calculations employ Slater–Koster parameter sets from the matsci libraries as implemented in the DFTB + package. With the relaxed, isolated molecular and crystalline structures under investigation, modeling the adsorbed structures is initiated. One places each isomer at 3.0 Å from the α-Fe(110) surface. Two proposed configurations initiate the simulation with the planar structure and two perpendicular structures on the surface. We set the first two layers of the outermost surface under investigation free to relax while keeping the two innermost layers fixed. After relaxing the structures of each complex, we calculate various properties. The first is the adsorption energy (*E*_ads_) in the gas phase

1 $$E_{ads}=E_{comp}-E_{surf}-E_{inh}$$

where *E*_comp_, *E*_surf_, *E*_inh_ correspond to the energy of the complex formed (surface + inhibitor), the surface, and the inhibitor studied, respectively, in the adsorption configuration. According to this definition, the more negative the adsorption energy, the greater the energetic stability of the created complex.

Another significant calculation performed is the charge density difference (Δρ) in the gas phase

2 $$\Delta \rho =\rho_{compl}-\rho_{surf}-\rho_{inh}$$

where ρ_comp_, ρ_surf_, ρ_inh_ correspond to the charge density of the complex formed (surface + inhibitor), the surface and the inhibitor investigated in the adsorption configuration, respectively. According to this definition, the charge density difference demonstrates the redistribution of charge between the adsorbent and the adsorbate during intermolecular interactions.^30^

In general, calculations for corrosion-inhibitor systems and the protected surface are performed in the absence of water using quantum methods. However, to better approximate the structure of the natural experimental system, 20 water molecules are incorporated into the calculations. The explicit inclusion of 20 water solvent molecules is not intended to reproduce bulk solvation or a complete three-dimensional hydration shell. Instead, this model represents a first interfacial hydration layer at the α-Fe(110) surface, the region most relevant to inhibitor adsorption and corrosion inhibition. The chosen number of water molecules ensures sufficient surface coverage to enable hydrogen bonding with both the metal surface and the adsorbed inhibitor while preserving computational tractability within the SCC-DFTB framework. It is important to note that all hydrated simulations use the same number and initial distribution of water molecules for both enantiomers, enabling a consistent comparative analysis. The relative stability trends and the conclusions reported here are robust with respect to the chosen hydration model. One calculates the corresponding adsorption energy (*E*_ads–water_) on the complex system containing the water molecules in the following way

3 $$E_{ads-water}=E_{comp+water}-\left(E_{inh+water}+E_{surf+water}\right)+E_{water}$$

where *E*_comp+water_, *E*_inh+water_, *E*_surf+water_, *E*_water_ refer to the energy of the structure of complex + water molecules, inhibitor + water molecules, surface + water system, and water molecules at the adsorption configurations, respectively.

The calculation of the charge density difference for this system is as follows

4 $$\Delta \rho_{water}=\rho_{comp+water}-\left(\rho_{inh+water}-\rho_{surf+water}\right)+\rho_{water}$$

We visualize the structures involved in this work using the VMD package.^31^

## 3 Results and Discussion

### 3.1 Molecular Properties

After obtaining the relaxed molecular enantiomer structures, we begin characterizing their electronic structures. We calculate the difference between the highest occupied molecular orbital (HOMO) and the lowest unoccupied molecular orbital (LUMO), known as the HOMO–LUMO gap. Depending on the positions and energy differences of the Frontier orbitals, the inhibitor donates or receives charge to or from the surface, becoming more reactive and enabling greater corrosion inhibition.^32,33^ The HOMO–LUMO gap for both isomers is calculated to be 2.98 eV. The HOMO–LUMO gap indicates that the molecule can both donate and accept electrons, which is often associated with adsorption reactivity in corrosion-inhibition studies.^32^ Also, the calculated HOMO–LUMO gap for both carvone enantiomers is identical, reflecting their mirror-image electronic structures in the gas phase. Thus, the HOMO–LUMO gap of the investigated molecules should not be interpreted as a direct predictor of inhibition efficiency since no intrinsic electronic asymmetry exists between the two investigated enantiomers. The observed difference in inhibition performance cannot be attributed solely to Frontier-orbital energetics.

In fact, it must arise from molecular chirality’s constraints on the three-dimensional orientation of functional groups during adsorption. The stereochemical arrangement around the chiral center governs the spatial accessibility of the carbonyl oxygen and the π-electron system toward surface atoms. It dictates the adsorption geometry, orbital overlap, and surface coverage after relaxation. Therefore, the inhibition efficiency will be controlled by chirality-induced differences in adsorption configuration and surface–molecule coupling rather than by the electronic reactivity of the isolated molecular inhibitors.

### 3.2 Adsorption of Inhibitors on the α-Fe(110) Surface

In the calculations of the α-Fe(110) surface, the monoterpene molecules are initially positioned 3.0 Å from the surface in both the parallel and vertical directions. Then, the inhibitor/surface complexes are relaxed in the gas phase. Figure 2 and 3 represent the relaxed configurations of adsorbent–adsorbate pairs formed by the studied isomers and the α-Fe(110) surface.

**Figure 2:**
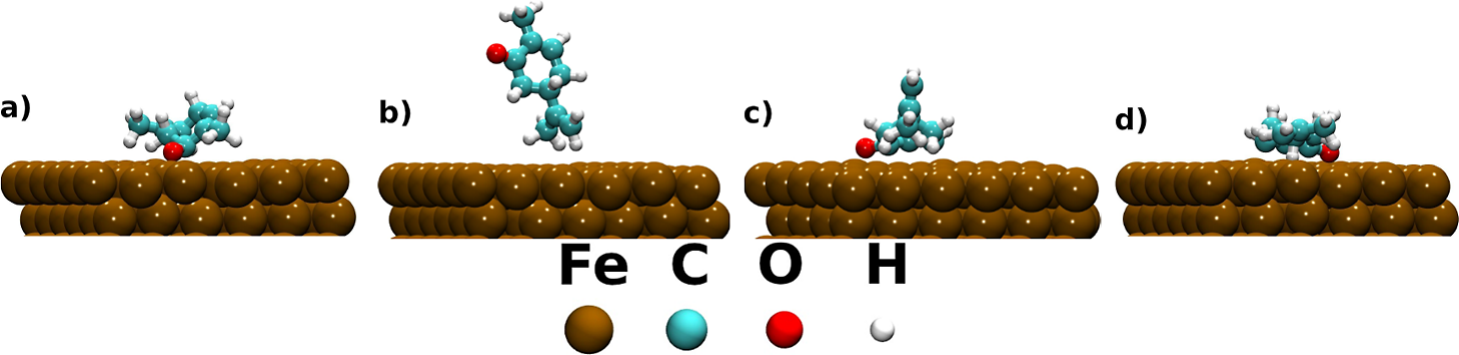
Relaxed adsorption configurations of scar on the top of the α-Fe(110) surface. (a) The most stable configuration has the isomer nearly parallel to the surface, allowing the carbonyl oxygen to anchor efficiently. (b) A less stable configuration shows the inhibitor/surface distance of 2.52 Å. (c) Another less stable configuration has a short Fe–O distance of 1.96 Å but is less stable than configuration (a). (d) A moderately stable configuration has an unfavorable lateral alignment of the oxygen atom with the surface.

Figures 2 and 3 display the relaxed adsorption configurations of scar and rcar on the α- Fe(110) surface. Tables 1 and 2 summarize the corresponding adsorption energies, charge transfer, and minimum green inhibitor–surface distances. In all the calculated configurations, the carbonyl oxygen atom plays a central role in anchoring the inhibitor molecule to the iron surface, serving as an essential adsorption site.

**Figure 3:**
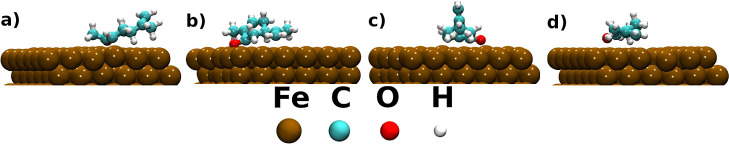
Relaxed adsorption configurations of rcar on the top of the α-Fe(110) surface. (a) The strong adsorption configuration has an energy of −7.8 eV, characterized by a direct O–Fe interaction at a distance of 2.01 Å. (b) The most stable configuration features an almost planar geometry that optimizes the orbital overlap between the oxygen and surface atoms. (c) An additional strong adsorption configuration demonstrates significant charge transfer, albeit with a tilted geometry. (d) The weakest configuration exhibits a separation distance of 2.31 Å and negligible charge transfer.

**Table 1:** Adsorption Energy (eV), Charge Transfer (e), and Minimum Distance (Å) Formed between the Scar and the Surface Models

configuration	*E*_Ads_ (eV)	charge (e)	distance (Å)
(a)	–8.6	–0.27	1.89
(b)	–0.5	0.01	2.52
(c)	–2.5	–0.23	1.96
(d)	–6.2	0.35	2.06

**Table 2:** Adsorption Energy (eV), Charge Transfer (e), and Minimum Distance (Å) Formed between the Rcar and the Surface Models

configuration	*E*_Ads_ (eV)	charge (e)	distance (Å)
(a)	–7.8	–0.21	2.01
(b)	–8.1	–0.16	1.98
(c)	–7.8	–0.25	1.97
(d)	–4.8	0.06	2.31

For both investigated inhibitor isomers, strong adsorption is associated with geometries in which the molecule adopts a nearly planar or slightly tilted orientation relative to the surface because this orientation allows the carbonyl oxygen to point toward iron surface atoms. In contrast, configurations where the oxygen atom is oriented away from the surface or laterally displaced exhibit weaker adsorption, larger molecule–surface distances, and negligible charge transfer.

For the scar, the configuration shown in Figure 2(a) is clearly the most stable, i.e., with an adsorption energy of −8.63 eV. This adsorption energy is consistent with the strong chemisorptive interactions reported for efficient organic inhibitors in the DFTB framework.^14^ It also exhibits the shortest O–Fe distance, calculated as 1.89 °A, and the most significant charge transfer from the surface to the molecule. In this configuration, the scar lies nearly parallel to the α-Fe(110) surface. It allows the carbonyl oxygen to serve as an efficient anchoring center.

Figure 2(b,c) depict less stable configurations. In particular, the configuration shown in Figure 2(b) exhibits a large molecule–surface distance of 2.52 Å and nearly zero charge transfer from the molecule to the investigated surface. It indicates that the oxygen atom is oriented away from the surface and does not form a chemical bond. The last configuration, shown in Figure 2(d), although moderately stable, i.e., with an adsorption energy of −6.26 eV, demonstrates a less favorable lateral alignment of the oxygen atom with respect to the investigated surface. Instead of pointing toward surface atoms, the oxygen atom remains displaced from the optimal adsorption position. It results in a large molecule–surface separation of 2.06 Å (Table 1), compared with that of the most stable configuration. As a consequence, the direct O–Fe interaction is weakened.

For the rcar-containing geometries, the configurations shown in Figure 3(a–c) present strong calculated adsorption energies ranging from 7.81 to 8.10 eV. They are characterized by short minimum distances of 1.97–2.01 Å. In these calculated cases, the oxygen atom is positioned near surface atoms, forming a direct O–Fe interaction. The configuration shown in Figure 3(b), which is the most stable with an adsorption energy of −8.10 eV, corresponds to an almost planar geometry in which the carbonyl group is aligned toward the surface, maximizing orbital overlap between the O and surface atoms.

In Figure 3(c), it exhibits the most significant charge transfer, despite having an adsorption energy similar to the configuration shown in Figure 3(a). It indicates that although the local O–Fe interaction is strong in Figure 3(c), the more tilted geometry of the inhibitor molecular structure reduces the stabilization of the adsorbate–surface system. Conversely, in the last configuration (Figure 3(d)), one can note a much weaker adsorption energy, followed by a larger adsorbate–adsorbent separation distance and negligible charge transfer from the inhibitor to the surface.

The differences in adsorption energies observed can be attributed to the substantial intermolecular interactions and orientation that the corrosion inhibitors form with the metallic surface in this study.^34^ The adsorption energy value of the scar/α-Fe(110) system is comparable to that of extremely potent organic corrosion inhibitors such as 2-(2-((1*H*-benzo­[d]­[1,2,3]­triazol-1-yl)­methyl)-4,5-dihydro-1H-imidazole-1-yl)­ethan-1-ol,^35^ (*E*)-2-(4-isobutylphenyl)- N’-(4-methoxybenzylidene)­propanehydrazide,^36^ and hennotanic acid.^37^ The rcar/α-Fe(110) system exhibits intense adsorption energy, comparable to that of thymol and carvacrol on the α-Fe(110) surface.^14^

Developing a comprehensive understanding of the interaction occurring between the isomers and the metallic surface through the adsorption process, we have illustrated the projected density of states (PDOS) in Figure 4. The PDOS plots (Figure 4) indicate the emergence of inhibitor-derived C and O 2p contributions near the Fermi level following adsorption. These new states reflect hybridization between the molecular orbitals of the inhibitors and the Fe 3d bands. It confirms the formation of partially covalent Fe–O and Fe–C bonds. The scar isomer exhibits slightly higher intensity near the Fermi level than rcar. It also indicates stronger orbital coupling to the metal surface.^34,38^ The enhanced hybridization observed near the Fermi level indicates stronger electronic coupling between the inhibitor and the α-Fe(110) surface. Despite the fact that the macroscopic adhesion parameter, such as the work of adhesion, is not calculated, the combination of more negative adsorption energies, shorter Fe–O interfacial distances, and pronounced charge redistribution at the interface suggests the formation of a densely bound, electronically stable adsorbed inhibitor layer. These properties, within an atomistic framework, are associated with increased resistance to desorption and improved surface coverage, which are critical for effective corrosion inhibitors.^39,40^

**Figure 4:**
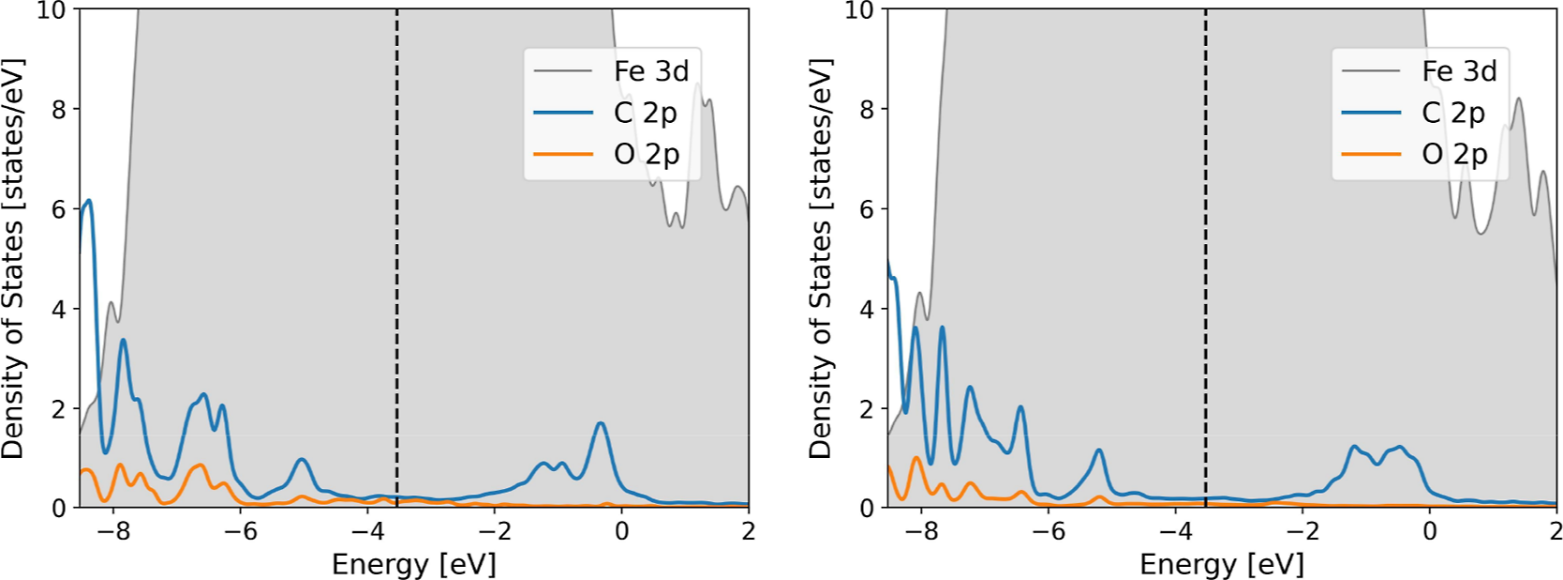
PDOS analysis for (left) scar and (right) rcar molecules adsorbed on α-Fe (110) surface.

Further information on the adsorption of the investigated monoterpenes, green corrosion inhibitors, to the α-Fe(110) surface is presented through an analysis of the charge density difference (CDD) (Figure 5). It is a valuable tool that can be used to visualize the local charge rearrangement resulting from the adsorption of green corrosion inhibitors, providing insight into how the adsorption affects the electronic properties of the investigated α-Fe(110) surface.^41^ Figure 5 shows an increase in charge density at the Fe–O bond interface (blue distribution) and a corresponding decrease at the investigated surface and inhibitors (yellow distribution), forming a chemical bond.^42^ The chemical bond formation through chemisorption (Figure 5) results in a redistribution of charges, generating a dipole moment that polarizes the surface.^43,44^ Based on Figure 5 and the previous results obtained in this work, it can be concluded that the inhibitors are chemically adsorbed on the iron surface via interactions with oxygen and carbon atoms.

**Figure 5:**
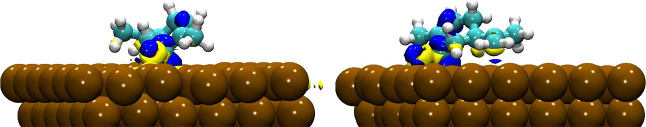
Charge density difference plots for scar (left) and rcar (right) adsorbed on the α- Fe(110) surface. Blue and yellow isosurfaces represent electron accumulation and depletion, respectively.

It is essential to perform an analysis employing reduced density gradient (RDG) techniques in conjunction with surface plots to visualize noncovalent interactions (NCI) and gain insights into their nature.^45^ The RDG scatter points are generated by calculating the product formed between the electron density and the sign of the second eigenvalue derived from the Hessian matrix.^46,47^

Figure 6 illustrates the isosurfaces of the reduced density gradient for the most stable systems. Upon examination, one can observe the details of the intermolecular interactions formed between the adsorbates and the adsorbent under study. The dispersion interactions manifest as a prominent green region distribution formed between the corrosion inhibitors and the investigated surface. A notable blue distribution is also observed, indicating strong interactions at the specific locations where each inhibitor binds to the surface. It highlights the distinct nature of the interactions, characterized by strong binding that contributes to inhibitor adhesion to the metal surface.

**Figure 6:**
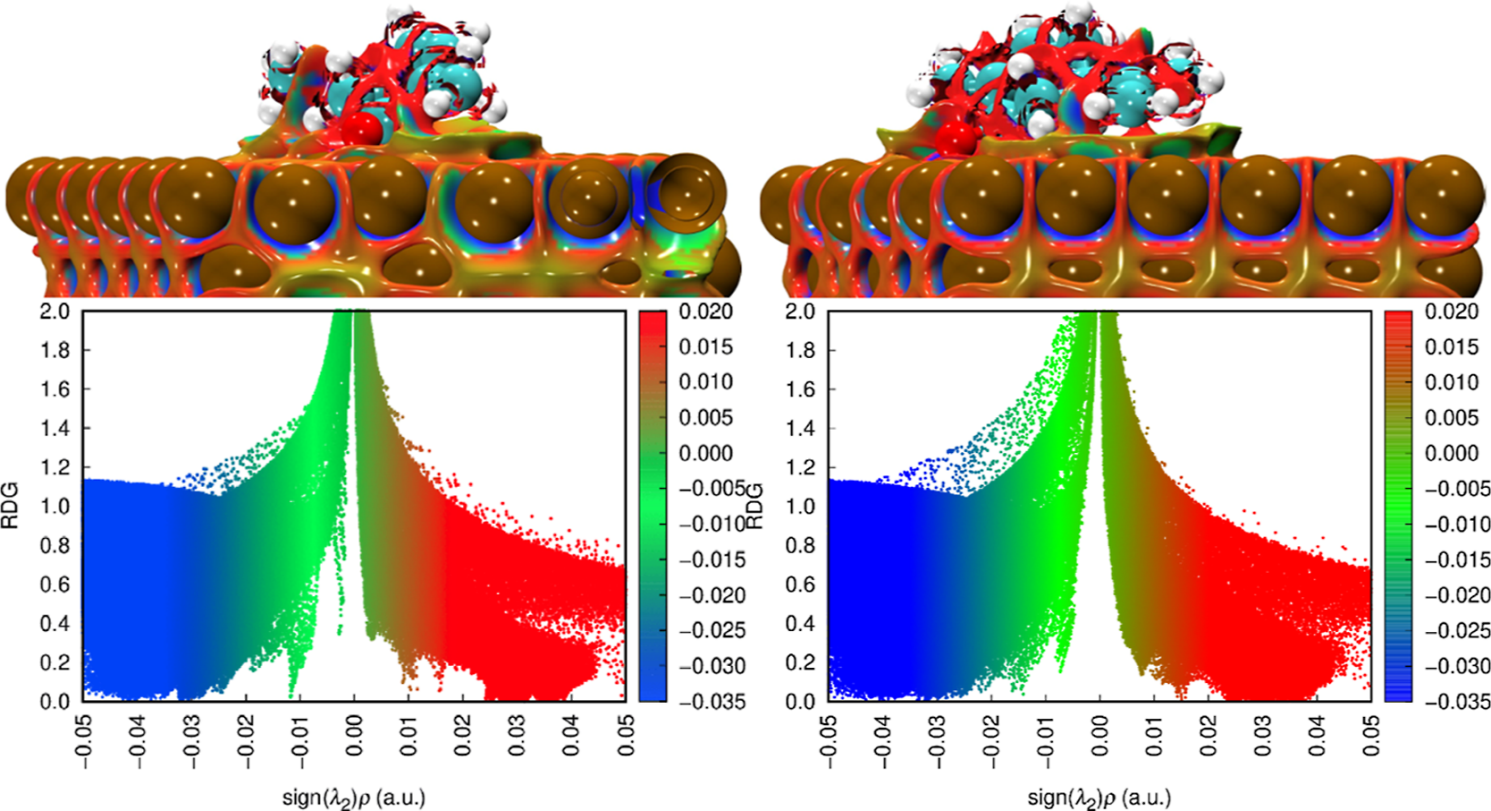
(Top) NCI plots and (bottom) RDG scatter plots for scar (left) and rcar (right) adsorbed on the α-Fe(110) surface.

## 4 Adsorption of Inhibitors on the Surface α-Fe(110) in the Presence of Water

Many theoretical calculations on the adsorption of corrosion-inhibitor molecules on metal surfaces have primarily been conducted without accounting for the aqueous environment in which these interactions typically occur.^48–50^ It limits our understanding of the performance of green inhibitors in real-world settings. Modeling studies should also account for the effects of water and other environmental factors to improve their relevance and accuracy. It would be valuable to gain a deeper understanding of how corrosion inhibitors interact with the α-Fe(110) surface for the intended applications, thus developing more effective formulations and new strategies for enhanced corrosion prevention.

Figure 7 illustrates the most stable forms of scar and rcar in adsorption configurations on the α-Fe(110) surface in the presence of water molecules. One can note that upon the introduction of water molecules, the fundamental adsorption mechanism remains unchanged. The computed adsorption energies demonstrate that hydration induces only minor geometric and energetic changes, preserving the dominant inhibitor–surface interactions. For scar, the adsorption energy changes only slightly from −8.63 eV in the gas phase to −8.70 eV in water, while rcar decreases from −8.10 to −7.75 eV. For the scar enantiomer, the molecule preserves its nearly parallel adsorption geometry, allowing water molecules to form hydrogen bonds with exposed polar sites without disrupting the Fe–O anchoring interaction. These hydrogen bonds act cooperatively, stabilizing the adsorbed configuration by reinforcing interfacial electrostatic interactions and improving the structural relaxation at the metal–inhibitor interface. It results in a slightly more favorable adsorption energy than in the gas phase. In contrast, the rcar isomer adopts a more tilted adsorption geometry due to steric constraints imposed by its chirality. In this configuration, water molecules form hydrogen bonds that partially compete with the Fe–O interaction. This competition induces a small lifting of approximately 0.15 Å of the molecule from the surface and weakens the overall adsorption stabilization, leading to a modest decrease in adsorption energy. Therefore, water plays a cooperative stabilizing role for scar, while exerting a softly competitive effect for rcar. It reflects the chirality-controlled balance between hydrogen bonding and metal–molecule coupling.

**Figure 7:**
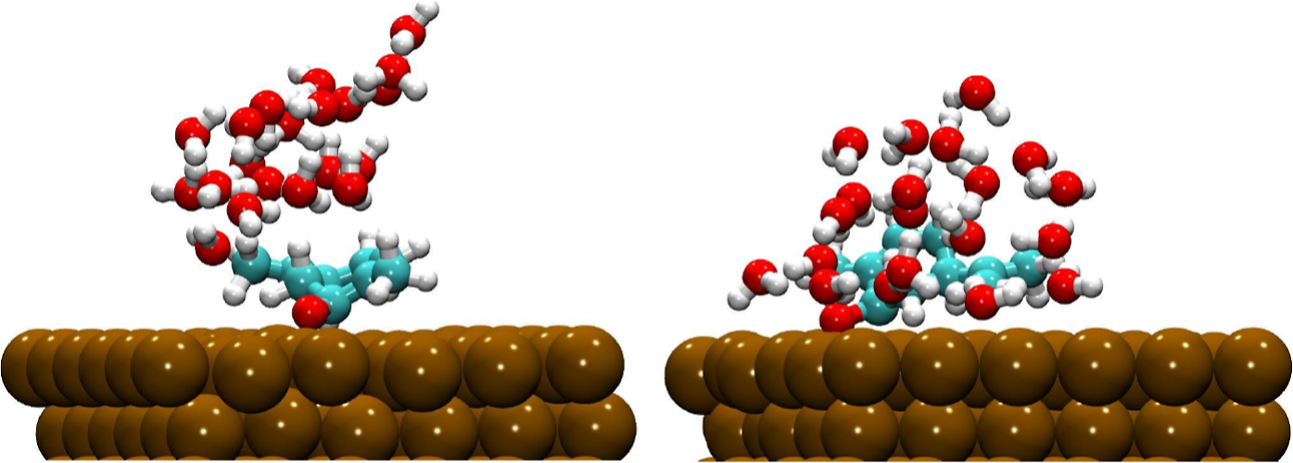
Relaxed adsorption configurations of the scar (left) and rcar (right) on the top of the α-Fe(110) surface, and 20 water molecules shown along the *a* axis.

Finally, Figure 8 illustrates the slight modification introduced by the interacting water molecules to the structures formed between the inhibitors and the metal surface under investigation. The CDD maps also remain essentially unchanged relative to the anhydrous environment, confirming that Fe–O and Fe–C interactions persist and that the fundamental electron-transfer mechanism between the inhibitor and the metal is maintained.

**Figure 8:**
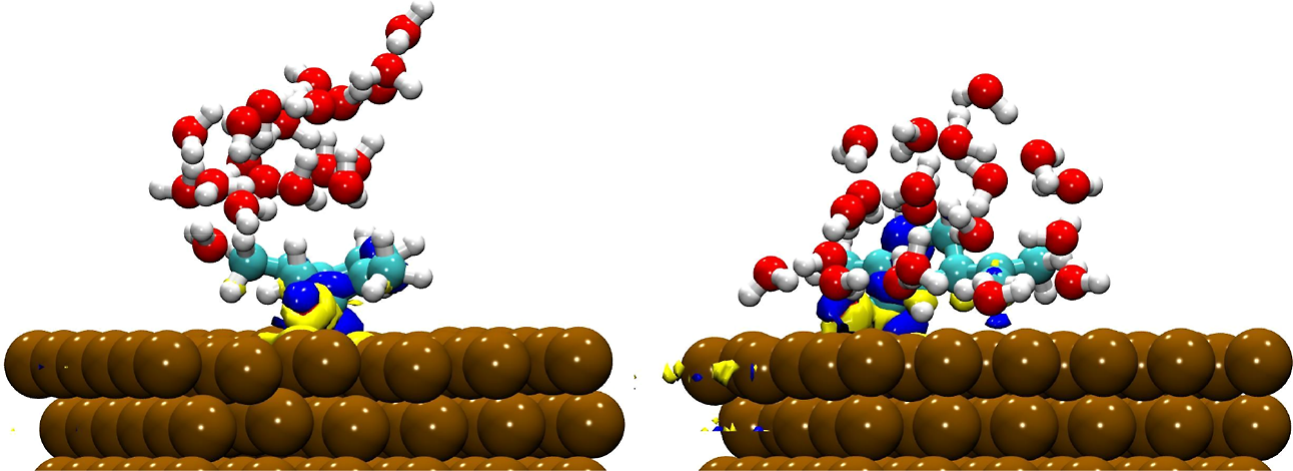
Charge density difference plots for scar (left) and rcar (right) adsorbed under the influence of water on the α- Fe(110) surface. Blue and yellow isosurfaces represent electron accumulation and depletion, respectively.

NCI/RDG analysis (Figure 9) indicates the formation of hydrogen bonds between water and the inhibitors functional groups, thereby stabilizing the adsorbed layer rather than disrupting it.

**Figure 9:**
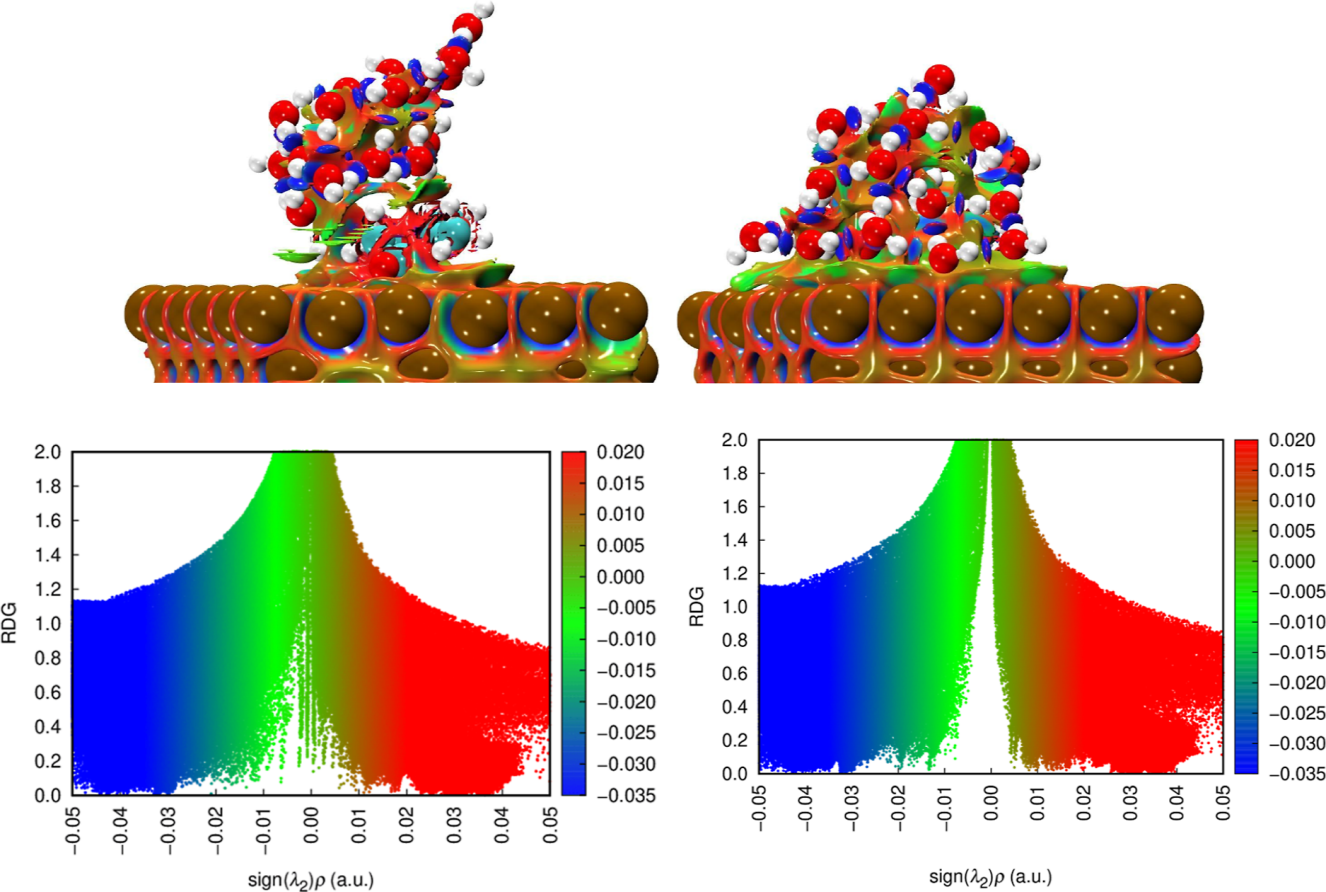
(Top) NCI plots and (bottom) RDG scatter plots for scar (left) and rcar (right) adsorbed on the α-Fe(110) surface under the influence of water.

The robust interaction between the metal surface under investigation and the isomeric organic inhibitors studied demonstrates remarkable stability, i.e., minimal reactivity upon contact with water molecules is noted. Organic inhibitors play a crucial role in providing a protective coating layer before solvent exposure for the duration of the intended engineering service. They have formed a barrier to protect the metal surface under study and remain intact in an aqueous environment.

## 5 Inhibition Mechanism

From an atomistic and electronic-structure perspective, the inhibition mechanism involves the simultaneous suppression of anodic metal dissolution and cathodic reduction reactions. One can note in Figure 10 that a schematic summary of this mechanism is provided. It is derived from the adsorption geometries, electronic structure, and charge-transfer characteristics obtained from the SCC-DFTB calculations.

**Figure 10:**
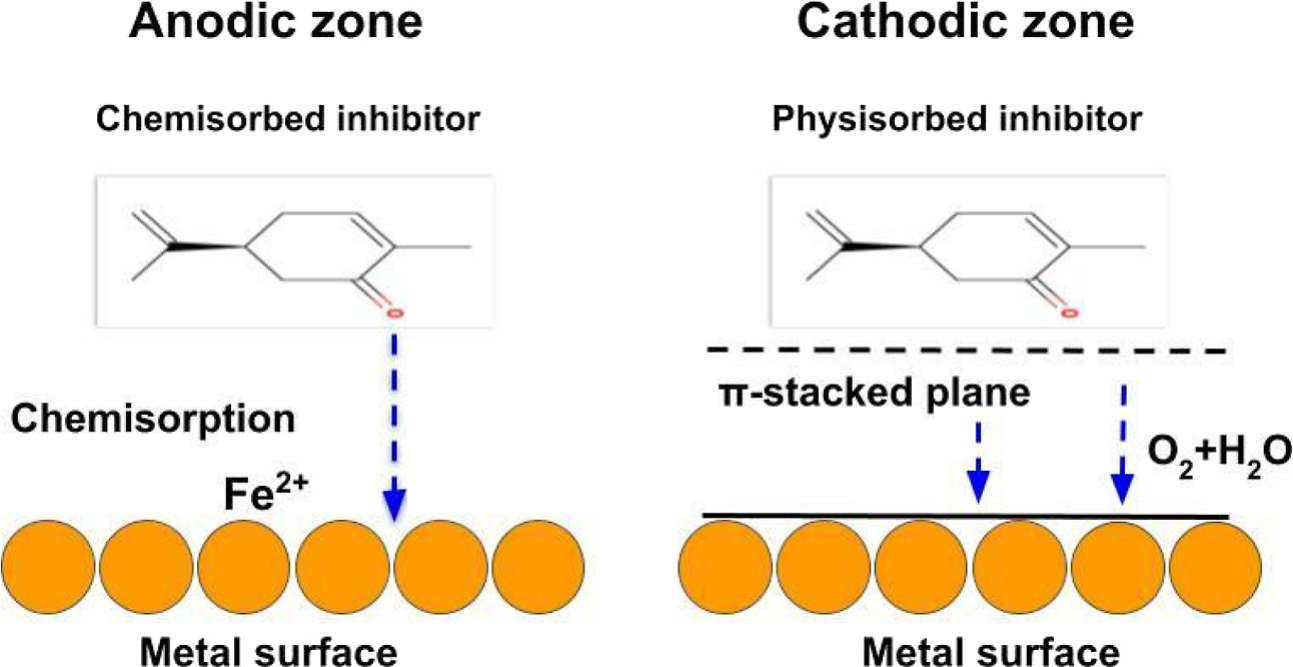
Proposed inhibition mechanism illustrating the adsorption of carvone molecules onto the anodic and cathodic active sites of the α-Fe(110) surface.

At anodic sites, both carvone enantiomers chemisorb on the α-Fe(110) surface primarily via the carbonyl oxygen and the π-electron system of the cyclohex-2-enone ring. This interaction is supported by considerable negative adsorption energies, short Fe–O distances, and pronounced charge redistribution at the interface, as revealed by CDD analysis. The charge transfer from the surface stabilizes the metal lattice and increases the activation barrier to iron dissolution, inhibiting anodic corrosion.^51^

At cathodic sites, the inhibition mechanism is governed by surface coverage and electronic blocking. The nearly parallel adsorption geometry adopted by the scar isomer enables more extensive surface coverage than the tilted configurations favored by rcar. This flatter geometry maximizes overlap between the inhibitor’s molecular orbitals and Fe 3d states, as evidenced by the stronger PDOS intensity near the Fermi level. The resulting hybridized electronic states and interfacial dipole formation hinder the charge transfer required for cathodic reactions.^51^

The adsorbed inhibitor layer acts as an electronic and steric barrier, i.e., it reduces the availability of active cathodic sites and suppresses electron flow between the metal surface and corrosive species.^51^ This blocking effect is more pronounced for scar due to its stronger adsorption, enhanced orbital coupling, and greater surface coverage. Thus, the combined anodic stabilization and cathodic charge-transfer suppression account for the superior inhibitory performance of the (*S*)-(+)-carvone isomer observed in the simulations.^51^

## 6 Conclusions

SCC-DFTB calculations are used in this work to investigate the corrosion-inhibition behavior of the chiral monoterpene isomers (*R*)-(-)-carvone and (*S*)-(+)-carvone on the α-Fe(110) surface. The results reported here highlight chirality as a key molecular descriptor governing adsorption geometry and electronic coupling on the α-Fe(110) surface, thereby determining inhibition efficiency.

Both organic isomers adsorb strongly on the investigated iron surface via a combination of chemisorption and physisorption, with the carbonyl oxygen serving as the primary anchoring site. The (*S*)-(+)-carvone isomer exhibits more favorable adsorption characteristics, i.e., more favorable adsorption energies, shorter Fe–O distances, and a flatter adsorption geometry compared to (*R*)-(-)-carvone. It enhances orbital hybridization between inhibitor C/O states and Fe 3d bands. It results in a more pronounced charge redistribution and the formation of a compact, adherent adsorbed inhibitor film.

The presence of an explicit aqueous environment during the calculations does not destabilize the inhibitor–surface complexes. It suggests that the inhibition mechanism remains effective under realistic corrosive conditions.

Carvone, an organic compound found in Amazon essential oils and known for its anticorrosive properties, shows promising trends that offer insights for future electrochemical studies. Our calculations suggest that the scar stereoisomer is a promising candidate for further experimental and electrochemical evaluation as a green corrosion inhibitor.
